# Estimating risk ratio from any standard epidemiological design by doubling the cases

**DOI:** 10.1186/s12874-022-01636-3

**Published:** 2022-05-30

**Authors:** Yilin Ning, Anastasia Lam, Marie Reilly

**Affiliations:** 1 Saw Swee Hock School of Public Health, National University of Singapore and National University Health System, Singapore, Singapore; 2 Duke-National University of Singapore Medical School, Singapore, Singapore; 3 School of Geography and Sustainable Development, University of St Andrews, St Andrews, United Kingdom; 4 Max Planck Institute for Demographic Research, Rostock, Germany; 5 Department of Medical Epidemiology and Biostatistics, Karolinska Institutet, Stockholm, Sweden

**Keywords:** Doubling-of-cases, Expanded data logistic regression, Log-binomial regression, Poisson regression, Relative risk, Weighted analysis

## Abstract

**Background:**

Despite the ease of interpretation and communication of a risk ratio (RR), and several other advantages in specific settings, the odds ratio (OR) is more commonly reported in epidemiological and clinical research. This is due to the familiarity of the logistic regression model for estimating adjusted ORs from data gathered in a cross-sectional, cohort or case-control design. The preservation of the OR (but not RR) in case-control samples has contributed to the perception that it is the only valid measure of relative risk from case-control samples. For cohort or cross-sectional data, a method known as ‘doubling-the-cases’ provides valid estimates of RR and an expression for a robust standard error has been derived, but is not available in statistical software packages.

**Methods:**

In this paper, we first describe the doubling-of-cases approach in the cohort setting and then extend its application to case-control studies by incorporating sampling weights and deriving an expression for a robust standard error. The performance of the estimator is evaluated using simulated data, and its application illustrated in a study of neonatal jaundice. We provide an R package that implements the method for any standard design.

**Results:**

Our work illustrates that the doubling-of-cases approach for estimating an adjusted RR from cross-sectional or cohort data can also yield valid RR estimates from case-control data. The approach is straightforward to apply, involving simple modification of the data followed by logistic regression analysis. The method performed well for case-control data from simulated cohorts with a range of prevalence rates. In the application to neonatal jaundice, the RR estimates were similar to those from relative risk regression, whereas the OR from naive logistic regression overestimated the RR despite the low prevalence of the outcome.

**Conclusions:**

By providing an R package that estimates an adjusted RR from cohort, cross-sectional or case-control studies, we have enabled the method to be easily implemented with familiar software, so that investigators are not limited to reporting an OR and can examine the RR when it is of interest.

## Background

The familiarity and wide adoption of logistic regression analysis for binary outcomes has resulted in the independent effect of a risk factor being most commonly reported as an adjusted odds ratio (OR) from logistic regression. The ease of communication and interpretation of a risk ratio (RR, also known as relative risk) is well recognized [1] and it is common for investigators to present and discuss the OR as an approximation to a RR for a rare outcome. However, each of these estimators comes with some consequences [2] and their advantages and disadvantages have been discussed extensively in the epidemiological literature. An important limitation of the OR, that is not shared by the RR, is the noncollapsibility that is the subject of ongoing discussion [3]. As a result of this property, the OR can vary across sub-groups defined by a variable unrelated to the exposure, which imposes limitations on its interpretation. Another disadvantage of the OR that is not shared by the RR is that it is sensitive to the choice of scale [4]. Thus there are situations where an adjusted RR can provide a better understanding of the data and research findings [5] and overcome the limitations of only reporting an OR [6]. Of particular concern in global public health is the misinterpretation of the OR as a RR, supporting exaggerated claims of the magnitude of associations [7].

If the underlying disease process follows a relative risk model, and not a logistic model, methods have long been available for estimating the RR from cohort or cross-sectional data: using log-binomial regression [8], or if this has convergence issues, Poisson regression [9] or Cox regression [10]. In the early years of case-control studies, a simple “correction” to the OR was proposed to yield a less biased estimate of the RR [11], but this was later shown to be biased in the presence of confounding [9]. A paper discussing eight methods of estimating the RR [12] from cohort or cross-sectional data presented an intriguing approach referred to as “doubling-the-cases”, motivated in the early 1980’s by Miettinen [13], where manipulation of the data enables the RR to be estimated using standard logistic regression. Assuming the outcome in the data is coded as 1 for cases and 0 for non-cases, the data set is expanded with an additional record for each case, in which the outcome is changed to 0, and a logistic regression analysis of this expanded data set provides an unbiased estimate of the RR. However, the naive standard error reported by the logistic regression is only valid for low incidence rates, and is otherwise biased upwards, representing the additional uncertainty that has been added to the data by having the same individual covariate profile associated with being both a case and a non-case. A robust sandwich estimator, first proposed in the early 1990s [14], corrects for the doubling of cases in the modified data, and has since been shown to perform well in simulation studies [12, 15]. However, statistical software packages do not provide an estimate of this standard error, so that a valid measure of precision is not easily available for the RR estimate. As a result of this computational challenge for cohort and cross-sectional studies, and the lack of methodology and software for case-control sampling, the simple and intuitive doubling-of-cases approach is absent from the standard tool-box of health researchers.

The early work that developed the robust standard error [14] demonstrated that the doubling-of-cases approach can also be applied to case-cohort data. Since the subcohort is a random sample of the whole cohort, it can be easily shown that the logistic regression of the expanded case-cohort data provides a valid estimate of the RR, and the prevalence can be recovered from the intercept using the subcohort sampling fraction. Unlike the subcohort in a case-cohort study, when a case-control sample is drawn from a cohort, these data are not representative of the larger cohort, resulting in the distortion of the estimate of RR (but not of the OR). However, if the sampling fractions are known, the cohort can be represented by up-weighting the observed data using sampling weights [16]. Since the doubling-of-cases approach uses the standard logistic regression model, it is straightforward to accommodate such sampling weights for valid estimation of the RR from case-control samples. However, additional work is required to incorporate the weights when correcting for the overestimation of variability due to the doubling of cases.

In this paper, we describe the doubling-of-cases approach in the cohort setting and then extend its application to the estimation of adjusted RR from case-control data, where the controls are selected either by random or stratified sampling. We derive an expression for the robust standard error and facilitate the use of the method by implementing it as an R package. We evaluate the performance of the approach using simulated data, and illustrate its application in the analysis of the effect of preterm birth on the risk of neonatal jaundice.

## Methods

### Doubling of cases in cohort studies

**Fig. 1:**
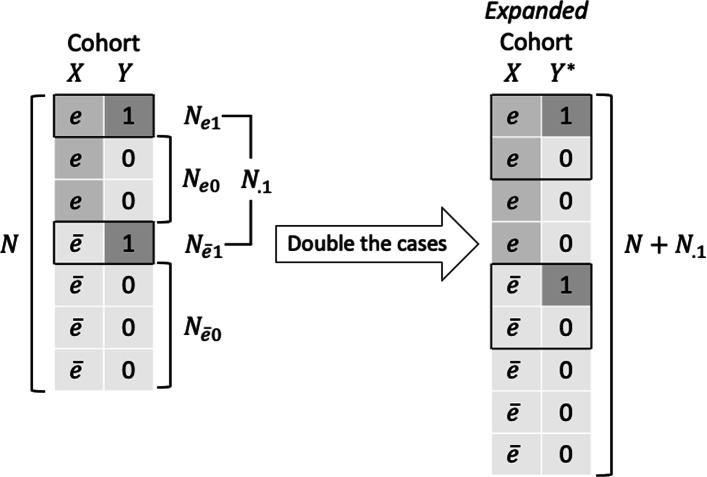
Doubling the cases in a cohort of *N* subjects, where *N*_.1_ subjects are cases. The first subscript indicates the exposure status (*e* for exposed and $\bar {e}$ for unexposed) and the second subscript indicates the outcome (1 for cases and 0 for non-cases). A dot (“.”) for either suffix denotes the total (i.e., no stratification on that variable)

To introduce the doubling-of-cases approach for estimating the RR, first consider a crude analysis using a cohort of *N* subjects, with a binary disease indicator *Y* (1 for cases and 0 for non-cases) and a binary exposure *X* (*e* for exposed and $\bar {e}$ for unexposed). As illustrated in Fig. 1, the doubling-of-cases approach involves expanding the cohort, by including each case twice, where the outcome on the second record is coded as a non-case. Such modification does not change the number of cases in the expanded cohort (where the outcome is denoted by *Y*^∗^), but increases the number of non-cases to *N* (see Fig. 1 and details in Table 1). Hence, the crude OR computed from the expanded cohort is identical to the RR from the original cohort.

**Table 1:** Equivalence of the crude RR computed from a cohort of *N* subjects and the crude OR computed from the expanded cohort with *N*+*N*_.1_ records, where $N_{.1} = N_{e1} + N_{\bar {e}1}$ is the total number of cases in the original cohort

**A) Cohort**	*Y*=1	*Y*=0	Total	Prevalence	Crude RR
*X*=*e*	*N*_*e*1_	*N*_*e*0_	*N*_*e*._=*N*_*e*1_+*N*_*e*0_	*p*_*e*_=*N*_*e*1_/*N*_*e*._	$RR = p_{e} / p_{\bar {e}}$
$X = \bar {e}$	$N_{\bar {e}1}$	$N_{\bar {e}0}$	$N_{\bar {e}.} = N_{\bar {e}1} + N_{\bar {e}0}$	$p_{\bar {e}} = N_{\bar {e}1} / N_{\bar {e}.}$	
**B) Expanded cohort**	*Y*^∗^=1	*Y*^∗^=0		Odds	Crude OR
*X*=*e*	*N*_*e*1_	*N*_*e*._		$odds^{*}_{e} = N_{e1} / N_{e.}$	$OR^{*} = p_{e} / p_{\bar {e}}$
$X = \bar {e}$	$N_{\bar {e}1}$	$N_{\bar {e}.}$		$odds^{*}_{\bar {e}}= N_{\bar {e}1} / N_{\bar {e}.}$	

#### Mantel-Haenszel OR from expanded cohort

In the presence of an additional categorical confounder, *Z*, the adjusted RR can be computed from the cohort using the Mantel-Haenszel approach, which is a weighted average of the RRs within each of the strata defined by *Z* [17]. Similarly, the Mantel-Haenszel OR from the expanded cohort is a weighted average of the ORs within each of the expanded strata (which are shown in Table 1 to be identical to the RRs in the original strata) using weights $w^{*k} = \left (N^{k}_{\bar {e}1} N^{k}_{e.}\right) / \left (N^{k} + N^{k}_{.1}\right)$ for the OR from the *k*-th expanded stratum, which differ from the weights used to compute the Mantel-Haenszel RR [17]: $w^{k} = \left (N^{k}_{\bar {e}1} N^{k}_{e.}\right) / N^{k}$ for the RR from the *k*-th stratum. It will be shown below that both the Mantel-Haenszel RR and the expanded data Mantel-Haenszel OR are estimating the same underlying parameter, the true adjusted RR.

#### Logistic regression of expanded cohort

The doubling-of-cases approach in regression analysis of cohort and case-cohort studies was first described in 1993 [14], and more recently was referred to as *expanded data logistic regression* [15]. Here we will briefly describe the approach by generalising the expanded data Mantel-Haenszel OR introduced above.

Assume the following relative risk log-binomial regression model for the probability of being a case for an individual with exposure *X* in stratum *Z*:

1 $$  \ln Pr(Y = 1 \mid X, Z) = \alpha + \beta X + \gamma Z,  $$

where exp*β* represents the adjusted RR (with adjustment for *Z*) [18–21]. When the cohort is expanded by doubling the cases, the prevalence in each exposure group in the original cohort becomes the odds in that exposure group in the expanded cohort (see Table 2). Hence, a log-linear model for the prevalence in the original cohort gives rise to a log-linear model for the odds, i.e., a logistic regression model, in the expanded cohort:

2 $$  \ln \frac{Pr(Y^{*} = 1 \mid X, Z)}{1 - Pr(Y^{*} = 1 \mid X, Z)} = \alpha + \beta X + \gamma Z,  $$

**Table 2:** Equivalence of the adjusted RR assessed in a log-binomial regression model of the original cohort with *N* subjects and the adjusted OR assessed in a logistic regression model of the expanded cohort with *N*+*N*_.1_ records, where $N_{.1} = N_{e1} + N_{\bar {e}1}$ is the total number of cases in the original cohort

**A) Cohort**	Expected *Y*=1	Expected *Y*=0	
*X*=*e*	*N*_*e*._ exp{*α*+*β*+*γZ*}	*N*_*e*._(1−exp{*α*+*β*+*γZ*})	
$X = \bar {e}$	$N_{\bar {e}.} \exp {\{\alpha + \gamma Z\}}$	$N_{\bar {e}.} (1 - \exp {\{\alpha + \gamma Z\}})$	
**B) Expanded cohort**	Expected *Y*^∗^=1	Expected *Y*^∗^=0	Odds
*X*=*e*	*N*_*e*._ exp{*α*+*β*+*γZ*}	*N*_*e*._	exp{*α*+*β*+*γZ*}
$X = \bar {e}$	$N_{\bar {e}.} \exp {\{\alpha + \gamma Z\}}$	$N_{\bar {e}.}$	exp{*α*+*γZ*}

which estimates the same regression coefficients as the log-binomial regression model in Eq. (1). The robust sandwich-type standard error (SE), derived by the same authors [14] to correct for this overestimation, is described in the next section.

#### Robust Sandwich-type SE for expanded data logistic regression

It can be readily seen from Table 2 that the probability of the modified outcome being 1 in the expanded cohort is:

3 $$  p^{*} = Pr(Y^{*} = 1 \mid X, Z) = \frac{Pr(Y = 1 \mid X, Z)}{1 + Pr(Y = 1 \mid X, Z)}.  $$

For the relative risk regression model defined in Eq. (1), the following pseudo log-likelihood was used [14] for estimating the regression coefficient, *β*, and its variability:

4 $$  {}\begin{aligned} l &= \sum_{i = 1}^{N} \{Y_{i} \ln(p^{*}_{i}) + \ln(1 - p^{*}_{i})\} \\ &= \sum_{i = 1}^{N} \{[Y_{i} \ln(p^{*}_{i}) + (1 - Y_{i}) \ln(1 - p^{*}_{i})] + Y_{i} \ln(1 - p^{*}_{i})\}, \end{aligned}  $$

where the subscript *i* indicates the *i*-th subject in the *original* cohort of *N* subjects. This pseudo log-likelihood is exactly the log-likelihood of the logistic regression of the expanded cohort, where the first component (in the square brackets) represents the regular log-likelihood contribution from the *N* subjects in the cohort, and the second component corresponds to the additional ‘non-cases’ created by doubling the cases. Hence, the regular maximum likelihood estimate from logistic regression analysis of the expanded data provides a valid estimate for *β*= ln(*RR*).

To describe the robust sandwich-type SE that was proposed [14] for the estimated ln(RR), it is useful to introduce a column vector to collectively denote the covariates observed from the *i*-th subject in the original cohort: ***x***_*i*_=(1,*X*_*i*_,*Z*_*i*_)^*T*^, where the first element corresponds to the intercept term in Eq. (1). The components in constructing the sandwich-type SE are derived from the following first-order derivative of the pseudo log-likelihood, *l*:

5 $$  U = \sum_{i = 1}^{N} U_{i} = \sum_{i = 1}^{N} \left\{Y_{i}\left(1 - p^{*}_{i}\right) - p^{*}_{i}\right\} \boldsymbol{x}_{i}^{T} = \sum_{i = 1}^{N} r^{*}_{i} \boldsymbol{x}_{i}^{T},  $$

where $r^{*}_{i} = Y_{i}(1 - p^{*}_{i}) - p^{*}_{i}$ is derived from the error terms (i.e., the difference between the observed outcome and the estimated probability) from the logistic regression of the expanded cohort. For a case in the original cohort, where $Y_{i} = 1, r^{*}_{i} = (1 - p^{*}_{i}) + (- p^{*}_{i})$ is the summation of the error terms corresponding to the two records in the expanded cohort, one as a case and the other coded as a non-case but with the same covariates (and hence the same probability $p^{*}_{i}$). For a non-case where $Y_{i} = 0, r^{*}_{i} = - p^{*}_{i}$ is the error term corresponding to the single record in the expanded data for this subject.

The proposed robust covariance matrix for the regression coefficients, (*β*,*γ*)^*T*^ is then:

6 $$  V = H_{1}^{-1} H_{2} H_{1}^{-1},  $$

where $H_{1}^{-1}$ is the inverse of the hessian matrix of *l*, estimated by the naive covariance matrix from the logistic regression of the expanded cohort, and *H*_2_ is the covariance matrix of *U*, estimated by:

7 $$  \hat{H}_{2} = \sum_{i = 1}^{N} \hat{U}_{i} \hat{U}_{i}^{T} = \sum_{i = 1}^{N} \hat{r}^{*2}_{i} \boldsymbol{x}_{i}^{T} \boldsymbol{x}_{i},  $$

and the $\hat {r}^{*}_{i}$ terms are computed from the residuals of the expanded data logistic regression as described above.

### Doubling of cases in case-control studies

When a case-control sample is drawn from a cohort, the sample prevalence is solely dependent on the case:control ratio. However, a case-control sample can be regarded as “intentionally missing” data, and provided the sampling fractions are known, valid cohort estimates (including the RR) can be obtained by up-weighting the sample observations using inverse probability weights to “reconstruct” the cohort. It is common for all cases in the cohort to be sampled into the case-control study, and for controls to be matched to cases on one or more characteristics. In such studies, the weight is 1 for the cases and the weights for controls are calculated as the inverse of the sampling fraction of the non-cases within the matching strata. If controls are selected by simple random sampling, the weights are simply the inverse of the overall sampling fraction of non-cases in the cohort.

#### Weighted logistic regression of expanded case-control data

As a direct extension of expanded data logistic regression for estimating the RR in cohort studies, we propose a weighted logistic regression of expanded data from a case-control study. As before, each case in the case-control sample is doubled, but the analysis of the expanded data is conducted with a weighted logistic regression, where the weight of each individual in the expanded data is inherited from the sampling fractions that yielded the original case-control sample. Note that doubling of cases is a part of the analytical approach and does not affect the sampling of case-control data or the calculation of sampling fractions. Using similar arguments as for cohort data [14], we propose a robust sandwich-type SE for the estimate of the *β* parameter in the logistic regression model, i.e., the estimated ln(RR), and describe it in the next section.

#### Robust SE for expanded data weighted logistic regression of case-control data

Consider the analysis of a case-control sample of *n* subjects drawn from a cohort of size *N*. Assuming all cases and a simple random sample of controls are included, the sampling weight (denoted by *w*) of each case in this case-control sample is 1, and for each control it is the number of controls in the cohort divided by the number of sampled controls. For matched case-control samples, the sampling weights for controls are the ratios of available controls to sampled controls within each stratum defined by the matching factors. An unbiased estimate of ln(RR) can be obtained from this case-control sample by using the doubling-of-cases approach, provided the individual sampling weights are incorporated in the analysis. More specifically, the pseudo log-likelihood becomes a weighted pseudo log-likelihood:

8 $$  l_{w} = \sum_{i = 1}^{n} \{w_{i} Y_{i} \ln(p^{*}_{i}) + w_{i} \ln(1 - p^{*}_{i})\},  $$

which is the log-likelihood corresponding to the weighted logistic regression analysis of the expanded case-control sample. The first order derivative of *l*_*w*_ is:

9 $$  {}U_{w} = \sum_{i = 1}^{N} w_{i} U_{i} = \sum_{i = 1}^{n} w_{i} \{Y_{i}(1 - p^{*}_{i}) - p^{*}_{i}\} \boldsymbol{x}_{i}^{T} = \sum_{i = 1}^{n} w_{i} r^{*}_{i} \boldsymbol{x}_{i}^{T}.  $$

Following the derivation of Eq. (6) for cohort designs, we propose the following as a robust covariance matrix for the estimates from a weighted analysis:

10 $$  V_{w} = H_{w1}^{-1} H_{w2} H_{w1}^{-1},  $$

where $H_{w1}^{-1}$ denotes the inverse of the Hessian matrix of *l*_*w*_ and is estimated by the naive covariance matrix from the (weighted) logistic regression of the expanded case-control data, and *H*_*w*2_ is the covariance matrix of *U*_*w*_, estimated by:

11 $$  \hat{H}_{w2} = \sum_{i = 1}^{n} w_{i}^{2} \hat{U}_{i} \hat{U}_{i}^{T} = \sum_{i = 1}^{n} (w_{i} r^{*}_{i})^{2} \boldsymbol{x}_{i}^{T} \boldsymbol{x}_{i}.  $$

### Simulation study

To evaluate our proposed estimator and robust SE for the RR from case-control data, we simulated a cohort consisting of *N*=1000 subjects, where 400 subjects were male (*Z*=1) and the remainder were female. To generate a confounding effect of sex, the probability of being exposed (*X*=1) was 0.4 for males and 0.2 for females and the outcome generated from the following log-binomial model:

12 $$  \ln P(Y = 1 \mid X, Z) = \alpha + \ln(RR) X + \ln(1.5) Z.  $$

The intercept term was assigned values corresponding to prevalence rates of approximately 10%, 20%, 30% and 40%. We considered true values of *RR*=1,1.25,1.5,2. For each simulated cohort, we implemented four designs: a 1:1 and 1:2 case-control ratio, each with controls selected randomly or matched on sex.

For the simulated cohort data, we estimated the RR using the log-binomial regression model (the true data-generating model), the expanded data logistic regression model, and other simple/naive estimators: the Mantel-Haenszel RR, expanded data Mantel-Haenszel OR, and the naive logistic regression model (where the estimated OR is viewed as an approximation for the RR). The case-control data was analysed by weighted logistic regression of the expanded data and by logistic regression of the original case-control sample. Although an unweighted logistic regression analysis with adjustment for matching factors is valid for estimating the OR of other covariates, we chose to perform a weighted analysis of matched case-control data to also enable valid estimation of the coefficients of the matching factors. The distributions of the estimates from the doubling-of-cases approaches over 2000 simulation cycles under each scenario were examined on boxplots, where they were compared to the estimates from the correct analysis (Mantel-Haenszel RR or log-binomial model) and the naive estimates. The performance of the method was evaluated by averaging the bias, empirical SE and robust SE, and computing the coverage of the (robust) 95% confidence interval, the type I error rate (when the true RR was 1) and power (when the true RR was not 1).

### Illustrative example

We analysed risk factors for neonatal jaundice in infants born to Swedish women between 1992 and 2002 [22]. From the singleton livebirths recorded by the Swedish Medical Birth Register during this calendar period, we excluded infants at risk of neonatal jaundice due to known maternal alloimmunisation or potential alloimmunisation due to a history of transfusion, resulting in 657,264 infants for analysis. In addition to the sex and prematurity of the infant, information was available for maternal age, body mass index (BMI), parity (nulliparous or multiparous) and smoking status. After excluding births with missing information on maternal BMI or smoking status, the final cohort consisted of 547,466 births. Maternal BMI was dichotomised at 25, and maternal age was dichotomised at 35 years. We assessed the association of neonatal jaundice with the six factors described above and the presence of an interaction between preterm birth and parity by analysing the full cohort and a 1:2 case-control sample matched on maternal age and the sex of the infant. The cohort data was analysed using naive logistic regression, log-binomial regression and expanded data logistic regression models. The matched case-control sample was analysed using weighted logistic regression and expanded data weighted logistic regression.

### Implementation

All analyses were performed using R (version 4.0.1). We implemented the expanded data (weighted) logistic regression model as an R package named *DoublingOfCases* (available from: https://github.com/nyilin/DoublingOfCases). The naive logistic regression model was implemented by the *glm* function with *family = binomial(link = “logit”)*, and for the weighted logistic regression, the inverse sampling weights were specified via the *weights* option. The log-binomial regression model was implemented by the *glm* function with *family = binomial(link = “log”)*.

## Results

### Simulation study

**Fig. 2:**
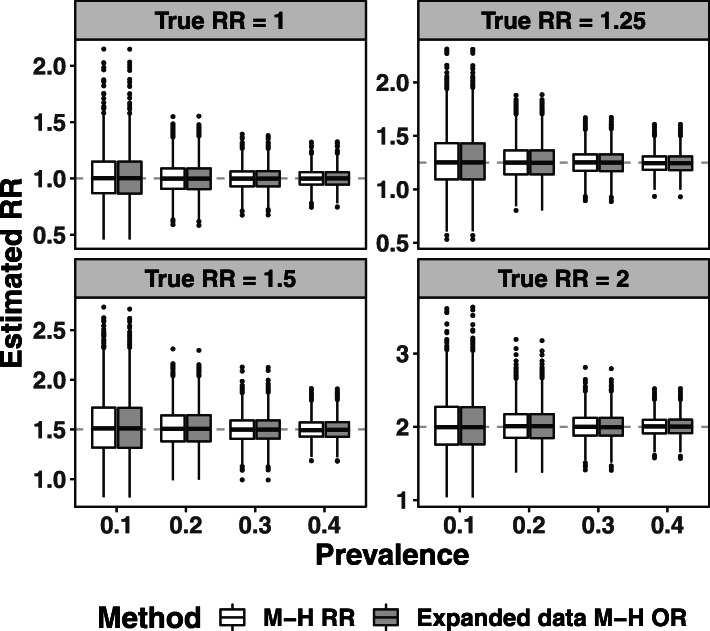
Estimated RR across 2000 simulations using different levels of prevalence and true RR values. Estimates were computed using the Mantel-Haenszel (M-H) RR (clear boxes) and expanded data M-H OR methods (shaded boxes)

**Fig. 3:**
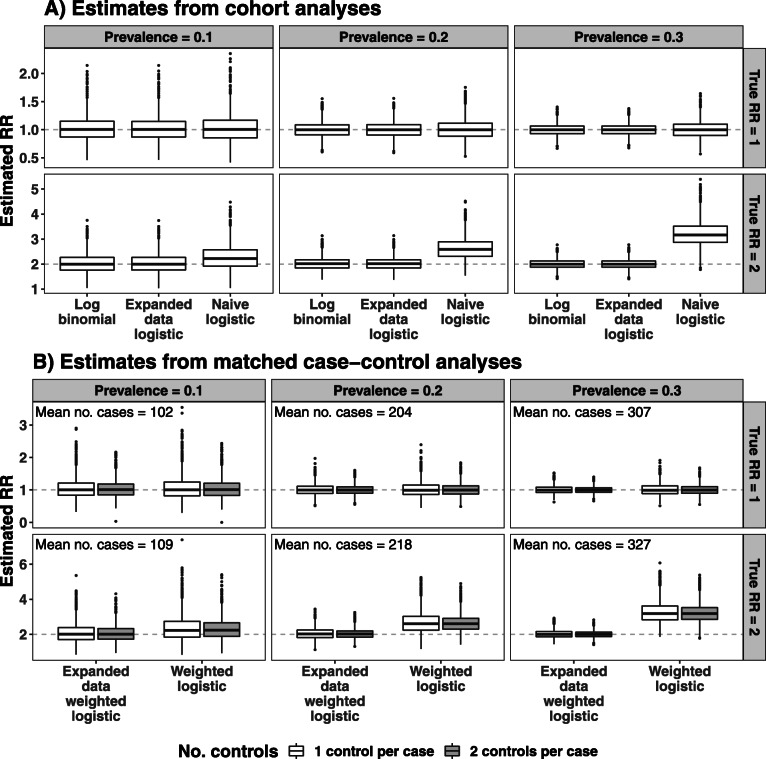
Estimated RR from expanded data logistic regression across 2000 simulations scenarios with different levels of prevalence and true RR values, compared to estimates from a naive logistic regression and the (true) log-binomial model. Estimates are presented for the full cohort data (panel A) and for matched case-control samples (panel B) with 1:1 (clear boxes) and 1:2 (shaded boxes) case:control ratio

For the simulated cohorts, the expanded data Mantel-Haenszel OR and expanded data logistic regression OR performed well, providing estimates similar to the Mantel-Haenszel RR and the log-binomial RR respectively, regardless of the prevalence in the cohort or the true value of the RR (see Figs. 2 and 3A). The bias in the naive OR increased as expected with larger values of RR and prevalence. Simulation scenarios with a prevalence rate of 40% and true RR of 1.5 or 2 approached the boundary of the parameter space of relative risk models, with the maximum event probability close to 0.80 and 0.95 respectively. The log-binomial regression model failed to converge in 2 and 1432 of the 2000 simulation cycles in these two scenarios respectively, but in the cycles where it converged, it provided valid estimates of the RR (see Appendix Table 5 for detailed simulation results).

**Fig. 4:**
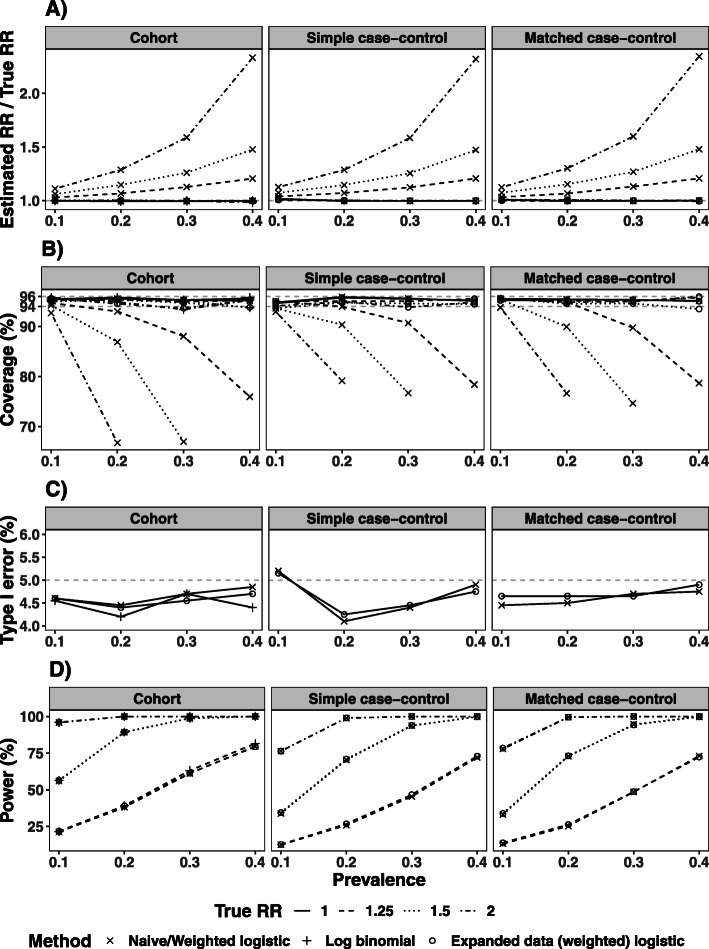
Ratio of the estimated and true RR (panel A) and the coverage (panel B), type I error (panel C) and power (panel D) of the RR estimated by naive and expanded data logistic regression analysis of case-control samples in simulation studies. 2000 simulation iterations were repeated in each simulation scenario. The results from log-binomial regression of the simulated cohort data are displayed for comparison: for a prevalence of 40%, this model failed to converge for 2 simulation cycles for RR=1.5 and 1432 cycles for RR=2, and these were excluded. Panel B excludes scenarios with RR > 1 where the naive/weighted logistic regression model had coverage lower than 50%

The “Cohort” column in Fig. 4 summarises the good performance of the expanded data logistic regression estimator in all simulation scenarios, with estimated RR close to the true value, coverage close to 95%, type I error close to 5% and power comparable to that of the log-binomial regression model. The robust SE of the estimated RR from the expanded data logistic regression model was similar to the empirical SE, and similar to the variability from the log-binomial regression model when the latter converged (see Appendix Table 5). The naive logistic regression model had a type I error close to 5% and power comparable with the expanded data logistic regression model in all scenarios, as might be expected. Although the estimated OR was a reasonable approximation to the RR (with small bias and coverage close to 95%) when the exposure had no effect (i.e., when RR = 1) or when the prevalence was low (10%), there was an increase in bias and decrease in coverage with increasing prevalence, especially when estimating a larger RR.

A similar performance was observed for the weighted logistic regression and expanded data weighted logistic regression models when applied to case-control data. Figure 3B presents the distributions of the RR estimates from 1:1 and 1:2 matched case-control studies, and the performance in terms of bias, coverage, Type I error and power are illustrated for random and matched 1:1 sampling in the second and third columns of Fig. 4 (details in Appendix Table 6).

### Illustrative example

A total of 21,441 (3.9%) of the infants in the cohort of 547,466 births were diagnosed with neonatal jaundice. The majority of these cases were firstborn infants, with only 3148 born to multiparous mothers. The crude OR associated with preterm birth was 28.0 but the crude RR was only 16.6, and the stronger association among multiparous mothers (crude OR=32.2 and crude RR=20.4) compared to nulliparous mothers (crude OR=23.4 and crude RR=13.1) suggested a possible interaction effect between these two factors. The large difference between the crude OR and RR suggests that the adjusted OR estimated from a naive logistic regression analysis would not be a reasonable approximation to the adjusted RR.

The log-binomial and expanded data logistic regression models provided similar estimates for the association of neonatal jaundice with each of the factors studied, except for a somewhat larger estimate for the association with overweight from the expanded data logistic regression model. Both models identified premature delivery as a strong risk factor for neonatal jaundice, with an estimated relative risk of approximately 13-fold among nulliparous mothers and 20-fold among multiparous mothers (see Table 3). Compared to infants of mothers with maternal BMI below 25, infants of overweight mothers (BMI ≥ 25) had an approximate 20%-26% higher risk of neonatal jaundice. Multiparity was associated with a decreased risk. Despite the low prevalence of the outcome in this population, the OR from a naive logistic regression model considerably overestimated the RR for preterm birth, almost by a factor of 2 for nulliparous mothers and 1.5 for multiparous mothers. Similar estimates were obtained by analysing the matched case-control sample using the weighted logistic and expanded data weighted logistic regression models, by incorporating the sampling weights (see Table 4).

**Table 3:** Adjusted ORs estimated using naive logistic regression, and adjusted RRs from log-binomial and expanded data logistic regression analysis, using data from a cohort study of neonatal jaundice. In addition to covariates in the table, estimates are adjusted for sex of infant, maternal age and smoking status

Variables	Naive logistic	Log-binomial	Expanded data
			logistic
	OR (95% CI)	RR (95% CI)	RR (95% CI)
Preterm: nulliparous	23.5 (22.4, 24.5)	12.9 (12.5, 13.3)	13.0 (12.6, 13.4)
Preterm: multiparous	32.5 (30.8, 34.2)	20.1 (19.4, 20.9)	20.4 (19.6, 21.2)
Overweight: BMI ≥ 25	1.30 (1.26, 1.34)	1.20 (1.17, 1.23)	1.26 (1.23, 1.30)
Multiparous	0.50 (0.48, 0.52)	0.51 (0.50, 0.53)	0.51 (0.49, 0.53)

**Table 4:** Adjusted ORs estimated from weighted logistic regression and adjusted RRs estimated from expanded data weighted logistic regression models, using data sampled in a 1:2 case-control design from the infant cohort, matched on infant sex and maternal age. In addition to covariates in the table, estimates are adjusted for smoking status

Variables	Weighted logistic	Expanded data
		weighted logistic
	OR (95% CI)	RR (95% CI)
Preterm: nulliparous	23.8 (22.7, 24.9)	13.1 (12.3, 13.9)
Preterm: multiparous	32.5 (30.9, 34.3)	20.5 (19.1, 21.9)
Overweight: BMI ≥ 25	1.32 (1.26, 1.39)	1.28 (1.23, 1.33)
Multiparous	0.50 (0.48, 0.52)	0.51 (0.49, 0.53)

## Discussion

Despite the attractive properties of the RR, there has been wide adoption of methods for estimating the OR, due in part to its mathematical and statistical properties, such as the reciprocity with respect to the choice of reference group [23] and the avoidance of predicted probabilities greater than 1. But the OR also has some unattractive properties not shared by the RR. Although the Mantel-Haenszel RR can be computed for simple tabular data, the more general log-binomial regression model for estimating an adjusted RR is not as widely known as the corresponding logistic regression model for estimating an adjusted OR. As a result of this familiarity, and the straightforward interpretation and ease of communication of the RR, investigators often present an adjusted OR as an approximation to the adjusted RR for rare outcomes. This has been further encouraged by articles in the medical literature that continue to present the OR as a feature of the case-control study design [24, 25], although there are methods of sampling that offer estimates of RR [26]. In addition to non-rare outcomes, there are other situations where the RR estimate may be useful or more appropriate, and a recent tutorial article on best practice encourages researchers to examine their results in more than one way [5] when there are valid alternatives. We have provided such an alternative, the doubling-of-cases approach, that is intuitively appealing and utilises the familiar logistic regression model after a simple modification to the data. Although it has been known for some time that this method provides a valid adjusted RR from cohort or cross-sectional data, the standard error of the estimate is not available from statistical software packages. In this paper, we first provided an introduction to this method in the context of cohort or cross-sectional data, and then extended the approach to data collected in a case-control design, deriving a robust estimate of the standard error. In contrast to the optional use of weighted logistic regression to improve precision or enable estimation of coefficients of matching factors [16, 27], a weighted analysis is *required* for valid estimation of a RR from case-control data. Where the case-control study has been implemented in a well-defined population or cohort, these weights are easily available from simple frequency distributions. To make the method accessible to data analysts, we have implemented it as an R package (available from https://github.com/nyilin/DoublingOfCases) that seamlessly estimates adjusted RRs from cohort, cross-sectional and case-control studies.

Using simulated data, we demonstrated that the expanded data weighted logistic regression of a case-control sample, with or without matching, produced similar estimates to the adjusted RR estimated from the full cohort. Our simulation studies also demonstrated the overestimation of a RR by the OR from a simple logistic regression model, even when the outcome is rare, especially for strong effects. In contrast, the weighted logistic regression model of the expanded data generated valid estimates for the RR, even for common outcomes. Our proposed robust SE for the RR estimated from case-control data performed well in estimating the variability of the adjusted RR.

In an application to neonatal jaundice, we found a positive association with preterm birth (which was stronger among multiparous mothers) and maternal overweight, and a negative association with multiparity, consistent with the literature [22, 28]. Although it is often assumed that the OR is a reasonable approximation for the RR when studying a rare outcome, this example demonstrated that the OR can considerably overestimate the RR of a rare event when assessing a very strong association: although the prevalence of the outcome (neonatal jaundice) in the cohort was only 3.9%, the adjusted ORs for preterm (23.5 and 32.5 among nulliparous and multiparous mothers, respectively) were considerably larger than the adjusted RRs estimated from log-binomial regression (12.9 and 20.1) or expanded data logistic regression (13.0 and 20.4).

**Fig. 5:**
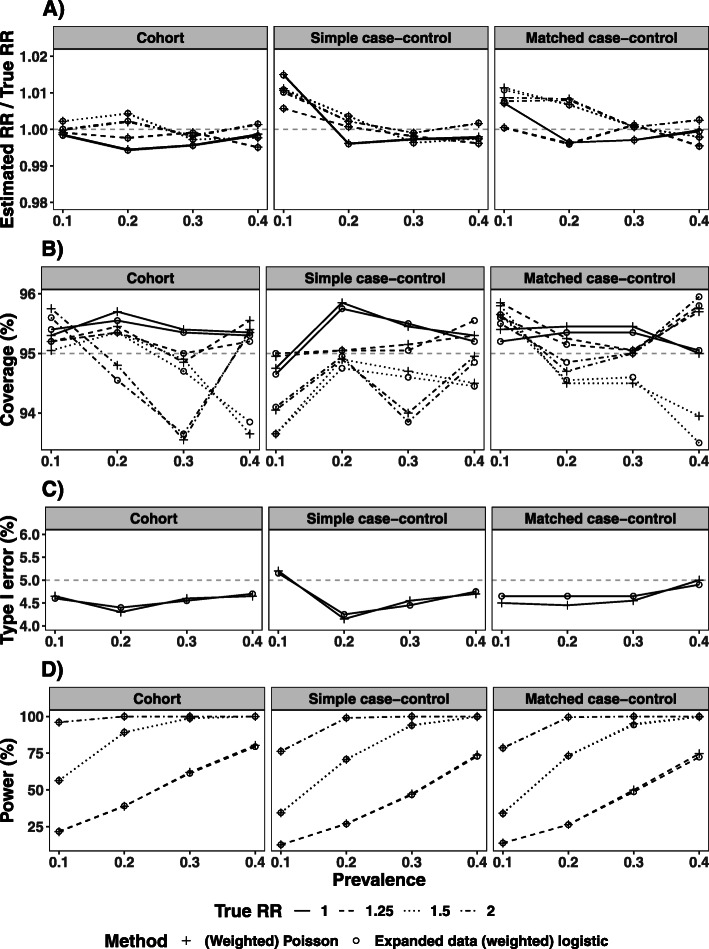
Ratio of the estimated to true RR (panel A) and the coverage (panel B), type I error (panel C) and power (panel D) of (weighted) Poisson regression of original data and (weighted) logistic regression of expanded data, from 2000 simulation iterations of each scenario

In our simulation study, we encountered a practical difficulty that is known in the implementation of log-binomial regression models in statistical software packages: the algorithm may fail to converge. In our simulation scenario of moderate effect (true RR=2) and high prevalence (40%), the log-binomial regression failed to identify valid starting values for the coefficients in more than 70% of the iterations. While this could be resolved by using crude RR estimates as starting values (data not shown), such issues may not be so easily overcome in practice. For example, Deddens and Petersen [29] created a simple numerical example with outcome *Y*=(0,0,0,0,1,0,1,1,1,1) and a single exposure *X*=(1,2,3,4,5,6,7,8,9,10), where the R implementation (via the *glm* function) failed to converge even when the true estimates were used as starting values. This difficulty, and sometimes inability, to reach convergence in maximising the likelihood of the log-binomial regression model, has been widely discussed in the literature [12, 15], and a computationally expensive approach to alleviate the problem has been made available in SAS [30]. An alternative approach that avoids convergence issues when estimating the RR is the Poisson regression model (with robust SE), which has a similar good performance to that of expanded data logistic regression when applied to cohort data [12, 15], or to case-control data that incorporates sampling weights (see Fig. 5 in Appendix). The Poisson regression model approximates the binomial distribution of the binary outcome using a Poisson distribution, whose statistical properties may not be familiar to many applied data analysts, making them reluctant to embark on such an analysis. In contrast, the doubling-of-cases approach is easily accessible as it leverages on the simple equivalence between the RR from the original data and the OR from the expanded data that is common to crude and adjusted analyses, and uses one of the most common analytical tools in epidemiology, the logistic regression model.

A potential practical limitation of the doubling-of-cases method for matched case-control data is that it is necessary to know the sampling fractions within the matching strata, as these are needed to enable the analysis to ‘reconstruct’ the background population/cohort from the case-control sample. The availability of this information will depend on whether the case-control study was conducted within a well-defined population, the nature and extent of the matching factors and the available data resources. Where a study is conducted using national or regional health registers, and cases and controls matched on basic demographic data (such as sex and age category), then the necessary information will be available from population statistics offices. The sampling fractions will also be known for studies that identify cases and controls from electronic medical records. However, the necessary data may be difficult or impossible to obtain for case-control studies that are implemented in the course of clinical work in low-resource settings with limited data infrastructure.

Another limitation of the doubling-of-cases approach, in common with Poisson regression, is the potential bias in the estimated ln(RR) when some subjects have estimated probabilities greater than or equal to 1. In the small numerical example from Deddens and Petersen [29] mentioned above, both the Poisson regression and the expanded data logistic regression had estimated probabilities larger than 1 for the 9th and 10th observations and both methods overestimated the RR to some extent: compared to the correct estimate (with 95% CI) of 1.23 (1.01 - 1.51), the Poisson regression with robust SE estimated the RR as 1.38 (1.13 - 1.70) and the expanded data logistic regression estimate was 1.44 (1.14 - 1.82). Although our illustrative example did not have large estimated probabilities (maximum 0.71), RR estimates are also known to be potentially biased when estimating a strong association with exposure [15], as occurred in the expanded data logistic regression analysis of the very strong association of prematurity with neonatal jaundice. Although the doubling-of-cases approach may result in some bias in the estimates of the RR in such settings, it can still be used by data analysts as a simple first approach. Large estimated probabilities may suggest that the log-linear assumption is inadequate, in which case the regression analysis should consider transformations of continuous covariates and/or interactions between covariates to more appropriately model the underlying data-generating mechanism.

## Conclusions

As a result of the method presented in this paper and the provision of a software package for its implementation, investigators can choose whether to report an adjusted OR or RR, or both, regardless of the study design. The method offers a simple and formal way of justifying the reporting of an adjusted OR as an approximate RR, regardless of the prevalence. Another important advantage is that it facilitates the comparison of findings to published RRs and the inclusion of estimates in meta-analyses that may be challenged by the mixed reporting of OR and RR.

## Appendix

Tables 5 and 6 present the detailed simulation results that were visualised in Fig. 4.

Figure 5 presents the results of a supplemental simulation study, in which each simulated cohort was analysed using Poisson regression, and each simple and matched case-control sample using weighted Poisson regression with inverse probability weighting. As illustrated in the Figure, the performance of the (weighted) Poisson regression was comparable with the doubling-of-cases approach in all scenarios investigated.

**Table 5:** Bias, empirical SE (Emp. SE), mean SE, coverage, type I error and power of ln*RR* from log-binomial and expanded data logistic regression analysis of simulated cohort, with 2000 simulation iterations in each scenario

Prevalence	True ln*RR*	Method	Bias	Emp.	Mean	Coverage	Type I/
				SE	SE		Power^1^
0.1	0	Log-binomial	-0.001	0.213	0.211	95.4	4.6
		Expanded data logistic	-0.002	0.214	0.211	95.4	4.6
	0.223	Log-binomial	-0.001	0.202	0.201	95.2	21.4
		Expanded data logistic	-0.001	0.202	0.201	95.2	21.7
	0.405	Log-binomial	0.002	0.194	0.194	95.0	56.5
		Expanded data logistic	0.002	0.194	0.194	95.2	56.4
	0.693	Log-binomial	0.000	0.185	0.185	95.8	96.0
		Expanded data logistic	0.000	0.185	0.185	95.6	96.0
0.2	0	Log-binomial	-0.005	0.136	0.139	95.8	4.2
		Expanded data logistic	-0.006	0.137	0.139	95.6	4.4
	0.223	Log-binomial	-0.002	0.131	0.131	95.6	39.1
		Expanded data logistic	-0.002	0.132	0.132	95.3	39.1
	0.405	Log-binomial	0.004	0.126	0.126	95.3	89.3
		Expanded data logistic	0.004	0.126	0.126	95.3	89.2
	0.693	Log-binomial	0.002	0.122	0.120	94.9	100
		Expanded data logistic	0.002	0.122	0.120	94.6	100
0.3	0	Log-binomial	-0.004	0.102	0.104	95.3	4.7
		Expanded data logistic	-0.004	0.103	0.105	95.3	4.6
	0.223	Log-binomial	-0.001	0.096	0.098	94.8	63.1
		Expanded data logistic	-0.001	0.098	0.099	95.0	61.3
	0.405	Log-binomial	-0.003	0.094	0.094	95.2	98.9
		Expanded data logistic	-0.003	0.095	0.095	94.7	98.7
	0.693	Log-binomial	-0.002	0.092	0.088	93.4	100
		Expanded data logistic	-0.002	0.093	0.089	93.7	100
0.4	0	Log-binomial	-0.001	0.081	0.082	95.6	4.4
		Expanded data logistic	-0.002	0.082	0.084	95.3	4.7
	0.223	Log-binomial	-0.005	0.074	0.076	95.8	81.6
		Expanded data logistic	-0.005	0.077	0.078	95.2	79.4
	0.405	Log-binomial^2^	-0.002	0.074	0.072	93.7	100
		Expanded data logistic	-0.002	0.076	0.073	93.8	100
	0.693	Log-binomial^3^	-0.017	0.067	0.067	95.2	100
		Expanded data logistic	0.002	0.068	0.068	95.3	100

^1^Values reported in this column are the type I error when true ln*RR*=0 and power otherwise.

^2^Based on 1998 simulation cycles where the log-binomial regression converged.

^3^Based on 568 simulation cycles where the log-binomial regression converged

**Table 6:** Bias, empirical SE (Emp. SE), mean SE, coverage, type I error and power of ln*RR* from expanded data weighted logistic regression analysis of simulated case-control data, with 2000 simulation iterations in each scenario

Design	Prevalence	True ln*RR*	Bias	Emp.	Mean	Coverage	Type I/
				SE	SE		Power^1^
Simple	0.1	0.000	0.015	0.299	0.289	94.7	5.2
case-		0.223	0.006	0.284	0.280	95.0	12.7
control		0.405	0.011	0.282	0.273	93.7	34.5
		0.693	0.010	0.269	0.26	94.1	76.4
	0.2	0.000	-0.004	0.174	0.178	95.8	4.3
		0.223	0.001	0.171	0.170	95.0	26.8
		0.405	0.004	0.165	0.164	94.8	70.9
		0.693	0.002	0.156	0.155	95.0	99.0
	0.3	0.000	-0.003	0.123	0.125	95.5	4.5
		0.223	-0.002	0.117	0.118	95.0	46.7
		0.405	-0.004	0.115	0.113	94.6	93.9
		0.693	-0.001	0.108	0.104	93.8	100
	0.4	0.000	-0.002	0.089	0.091	95.2	4.8
		0.223	-0.004	0.084	0.085	95.6	72.9
		0.405	-0.003	0.082	0.080	94.4	99.8
		0.693	0.002	0.072	0.072	94.8	100
Matched	0.1	0.000	0.007	0.287	0.283	95.2	4.7
case-		0.223	0.000	0.280	0.274	95.6	13.9
control		0.405	0.011	0.262	0.268	95.7	33.9
		0.693	0.008	0.256	0.257	95.5	78.4
	0.2	0.000	-0.004	0.173	0.175	95.3	4.7
		0.223	-0.004	0.167	0.168	95.2	26.3
		0.405	0.007	0.163	0.162	94.6	73.2
		0.693	0.008	0.158	0.154	94.8	99.5
	0.3	0.000	-0.003	0.121	0.123	95.3	4.7
		0.223	0.001	0.115	0.117	95.0	48.7
		0.405	0.001	0.113	0.112	94.6	94.2
		0.693	0.001	0.107	0.105	95.0	100
	0.4	0.000	-0.001	0.090	0.091	95.0	4.9
		0.223	-0.005	0.084	0.085	95.8	72.4
		0.405	-0.002	0.083	0.081	93.5	99.8
		0.693	0.003	0.074	0.075	96.0	100

^1^Values reported in this column are the type I error when true ln*RR*=0 and power otherwise

## Data Availability

Data sharing is not applicable to this article as no new data were created or analyzed in this study. The R package created for this application, named *DoublingOfCases*, is available for download from: https://github.com/nyilin/DoublingOfCases.

## Abbreviations

BMI: Body Mass Index
OR: Odds Ratio
RR: Relative Risk
SE: Standard Error
